# An Artificial Measurements-Based Adaptive Filter for Energy-Efficient Target Tracking via Underwater Wireless Sensor Networks †

**DOI:** 10.3390/s17050971

**Published:** 2017-04-27

**Authors:** Huayan Chen, Senlin Zhang, Meiqin Liu, Qunfei Zhang

**Affiliations:** 1 State Key Laboratory of Industrial Control Technology, Hangzhou 310027, China; chenhuayan@zju.edu.cn; 2 College of Electrical Engineering, Zhejiang University, Hangzhou 310027, China; slzhang@zju.edu.cn; 3 School of Marine Science and Technology, Northwestern Polytechnical University, Xi’an 710072, China; zhangqf@nwpu.edu.cn

**Keywords:** artificial measurements, energy-efficiency, target tracking, underwater wireless sensor networks

## Abstract

We study the problem of energy-efficient target tracking in underwater wireless sensor networks (UWSNs). Since sensors of UWSNs are battery-powered, it is impracticable to replace the batteries when exhausted. This means that the battery life affects the lifetime of the whole network. In order to extend the network lifetime, it is worth reducing the energy consumption on the premise of sufficient tracking accuracy. This paper proposes an energy-efficient filter that implements the tradeoff between communication cost and tracking accuracy. Under the distributed fusion framework, local sensors should not send their weak information to the fusion center if their measurement residuals are smaller than the pre-given threshold. In order to guarantee the target tracking accuracy, artificial measurements are generated to compensate for those unsent real measurements. Then, an adaptive scheme is derived to take full advantages of the artificial measurements-based filter in terms of energy-efficiency. Furthermore, a computationally efficient optimal sensor selection scheme is proposed to improve tracking accuracy on the premise of employing the same number of sensors. Simulation demonstrates that our scheme has superior advantages in the tradeoff between communication cost and tracking accuracy. It saves much energy while loosing little tracking accuracy or improves tracking performance with less additional energy cost.

## 1. Introduction

More than 70% of the earth’s surface is covered by seas and oceans. Seas and oceans are mysterious and charismatic to human beings because of the huge amount of unexploited resources. Underwater wireless sensor networks (UWSNs) technologies are developing gradually to enhance our abilities to discover resources in aquatic environments [1,2,3,4]. UWSNs are three-dimensional (3D) networks. The communication between underwater sensors relies on acoustic waves. UWSNs have a broad range of applications such as environmental monitoring, undersea exploration, disaster prevention, and distributed tactical surveillance, etc. We study the problem of accurately and energy-efficiently tracking a maneuvering target via UWSNs. UWSNs are the extending of wireless sensor networks (WSNs) which are applied to terrestrial environments [5,6,7]. One of the significant differences [8] between UWSNs and WSNs is the cost. Since underwater sensors need to work in the extreme underwater environment, they are much more expensive than terrestrial sensors. Underwater sensors use acoustic waves while terrestrial sensors use radio frequency waves. The energy consumption for communication between underwater sensors is higher than terrestrial sensors. Moreover, the sensors of UWSNs are battery-powered and it is impracticable to replace batteries when exhausted. This means that the battery life affects the lifetime of the whole network. Compared with the energy cost of sensing and processing, communication cost dominates the whole energy cost according to the energy model shown in [9]. Thus, in this paper, we improve the energy efficiency of target tracking by cutting down less helpful communications between local sensors and the fusion center.

This paper addresses the issue of implementing the tradeoff between the communication rate and target tracking accuracy. Local sensors need to figure out whether to send their information to the fusion center or not. This idea was inspired by some research about remote state estimation under communication constraints [10,11,12,13]. When a local sensor obtains a measurement about the target, it needs to figure out whether the measurement residual is large enough. A large measurement residual means the new measurement has enough value to be sent to the fusion center. If the measurement residual is larger than the threshold, the fusion center receives information from the local sensor and works as usual. If the measurement residual is smaller than the threshold, the fusion center receives nothing from the local sensor and generates an artificial measurement to approximate the unsent one, which makes full use of the information of the unsent measurement. Then, we derive the corresponding artificial measurements-based recursive form the filter. A preliminary version of the present paper appeared as a conference paper in [14]. The current version extends the conference version by providing an adaptive method for determining proper criteria which are used to tell local sensors whether their measurements have enough value to be sent to the fusion center. Moreover, a computationally efficient optimal sensor selection scheme is proposed to improve tracking accuracy on the premise of employing the same number of sensors.

The main contributions of this paper are threefold. First, we derive an artificial measurements-based filter, which has advantages in energy-efficiency. Second, in order to exploit advantages of our filter, we propose an adaptive method for determining proper criteria, which results in our artificial measurements based adaptive filter. Last, an optimal sensor selection scheme is proposed to further improve the energy-efficiency.

The rest of the paper is organized as follows. In Section 2, we discuss the related work in the area of target tracking in UWSNs. In Section 3, we formulate the problem and introduce some propaedeutic. In Section 4, we introduce our artificial measurements-based adaptive filter. In Section 5, we present our simulation results to verify our adaptive filter and discuss its characteristics. Finally, in Section 6, we provide the conclusions.

## 2. Related Work

Target tracking is a focused application for underwater defense systems. Intended targets to be tracked are unmanned underwater vehicles (UUVs) and submarines. As an emerging research interest, only a few works about target tracking in UWSNs can be found in the literature. In early work, a simple target tracking method utilizing only measurement information for 3D underwater is presented by Isbitiren et al. [15]. Based on the time of arrival of the echoes from the target after transmitting acoustic pulses from the sensors, the ranges of the nodes to the target are determined, and trilateration is used to obtain the location of the target. This method tracks the target only based on current measurements, which is adverse in terms of achieving high target tracking accuracy. In sparse networks, it results in tracking failure if not enough sensors are involved. In order to get better target tracking performance, Wang et al. [16] proposed an algorithm that combines the interacting multiple model (IMM) with the particle filter (PF) to cope with uncertainties in target maneuvers. To realize energy-effective target tracking, Yu et al. [17] provided an algorithm named wake-up/sleep (WuS) increasing energy efficiency of each sensor by using a distributed architecture. At each time step, WuS means waking up sensors that have an opportunity to detect the target and sending those that do not to sleep. However, it wastes energy by employing all candidate sensors without the survival of the fittest. Later, Zhang et al. [18] proposed an adaptive sensor scheduling scheme which saves energy by changing the sampling interval. The sampling interval is variable according to whether the tracking accuracy is satisfactory or not at each time step. The main distinction between this paper and the reference [18] is that they improve energy-efficiency from different dimensions. This paper focus on the tradeoff between communication rate and tracking accuracy at each time step, which is from a spatial dimension. Zhang et al. [18] focused on the tradeoff between sampling interval and tracking accuracy, which is from the temporal dimension. Recently, Zhang et al. [19] studied the effect of sensor topology on the target tracking in UWSNs with quantized measurements. They proposed a sensor selection method which selects the optimal topology by minimizing the posterior Cramer–Rao lower bound (PCRLB). This method improved the target tracking performance under the premise of employing the same number of sensors. However, the computation of PCRLB is complicated. In our work, we use the trace of predicted estimate covariance to select the optimal sensor group, which is more convenient than PCRLB.

## 3. Problem Formulation

This section formulates the problem of single target tracking via distributed UWSNs. The issues to be covered include system model, distributed fusion architectures and measurement residual-based sensor scheduling. For ease of reference, we list notations that will be used frequently in Table 1.

### 3.1. System Model

We consider the conventional target motion model, which is defined as

(1) $$X_{k}=F_{k}X_{k-1}+w_{k-1},$$

where $X_{k}$ denotes the target state (positions and velocities) at time *k*, $F_{k}$ is the state transition matrix at time *k* and $w_{k-1}$ is the process noise with zero-mean white Gaussian distribution $N(0,Q_{k-1})$.

UWSNs consist of *N* wireless acoustic sensors floating at different seawater layers. The positions of sensors in Cartesian coordinates are denoted by $s^{i}=\left(x_{i}^{s},y_{i}^{s},z_{i}^{s}\right),\;i=1,\ldots N$. Sensors measure the distance to the target by transmitting acoustic pulses (ping) and calculating the time-of-arrival (ToA) of the pings and echoes.

The measurement model of the sensor $s^{i}$ at time *k* is given by

(2) $$Z_{k}^{i}=h_{k}^{i}\left(X_{k}\right)+v_{k}^{i},$$

where $h_{k}^{i}\left(X_{k}\right)$ is the measurement function, and $v_{k}^{i}$ is the measurement noise with zero-mean white Gaussian distribution $N(0,R_{k}^{i})$. The measurement function is given by

(3) $$h_{k}^{i}\left(X_{k}\right)=\sqrt{{\left(x_{k}-x_{i}^{s}\right)}^{2}+{\left(y_{k}-y_{i}^{s}\right)}^{2}+{\left(z_{k}-z_{i}^{s}\right)}^{2}}.$$

where $\left(x_{k},y_{k},z_{k}\right)$ is the location of the target at time *k*. The corresponding Jacobian matrix $H_{k}^{i}$, which is a useful approximation technique from the well-known extended Kalman filter (EKF) of the measurement function $h_{k}^{i}\left(\cdot \right)$ is given by

(4) $$H_{k}^{i}\left(X_{k}\right)=\left[\begin{array}{c} \left(x_{k}-x_{i}^{s}\right)/d,0,\left(y_{k}-y_{i}^{s}\right)/d,0,\left(z_{k}-z_{i}^{s}\right)/d,0 \end{array}\right].$$

where $d=\sqrt{{\left(x_{k}-x_{i}^{s}\right)}^{2}+{\left(y_{k}-y_{i}^{s}\right)}^{2}+{\left(z_{k}-z_{i}^{s}\right)}^{2}}$ is the distance between the target and the sensor *i*.

### 3.2. Distributed Fusion Architectures

**Figure 1:**
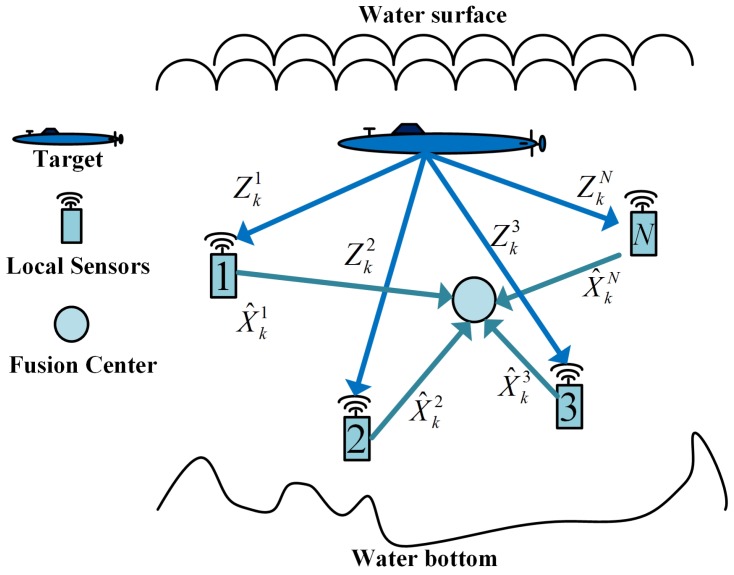
Conventional distributed fusion architecture for target tracking.

At the same time, different local sensors have different measurements about the same target. The information comes from different sensors must be fuse together to acquire more accurate estimates of target states. There are two types of fusion architectures, distributed fusion architectures and centralized fusion architectures. Distributed fusion architectures have advantages over centralized architectures in lower communications and processing costs. Therefore, distributed fusion architectures are preferential for application in resource-limited UWSNs. Figure 1 shows the normal structure of the distributed fusion system. Local sensors sample the measurements ($Z_{k}^{1},Z_{k}^{2},\cdots ,Z_{k}^{N}$) from the target periodically. Then, based on new measurements and past information (${\hat{X}}_{k-1}$), local sensors obtain local estimates (${\hat{X}}_{k}^{1},{\hat{X}}_{k}^{2},\cdots ,{\hat{X}}_{k}^{N}$) and transmit local estimates to the fusion center. Finally, the fusion center collects all local estimates and fuses them together to get the fusion estimate (${\hat{X}}_{k}$). The fusion estimate will be sent back to local sensors to predict future target states.

### 3.3. Measurement Residual-Based Sensor Scheduling

For the purpose of saving communication costs, we want local sensors to think carefully before sending their local estimates to the fusion center periodically as usual. If some local estimates have low values in updating the target state estimate, we should leave them at local sensors to reduce energy costs. The value of a local estimate can be measured by a measurement residual before we calculate the local estimate as

(5) $${\tilde{Z}}_{k}^{i}=Z_{k}^{i}-{\hat{Z}}_{k|k-1},$$

(6) $${\hat{Z}}_{k|k-1}=h_{k}^{i}\left({\hat{X}}_{k|k-1}\right),$$

where ${\tilde{Z}}_{k}^{i}$ is the measurement residual and ${\hat{Z}}_{k|k-1}$ is the predicted measurement. These make sense since the larger the measurement residual is, the larger the difference between the measurement updated estimate ${\hat{X}}_{k}$ and the predicted estimate ${\hat{X}}_{k|k-1}=F_{k}{\hat{X}}_{k-1}$ will be. A small measurement residual means it can change the predicted estimate ${\hat{X}}_{k|k-1}$ only a little, so the fusion center can simply keep the predicted estimate ${\hat{X}}_{k|k-1}$ or do some approximation.

**Figure 2:**
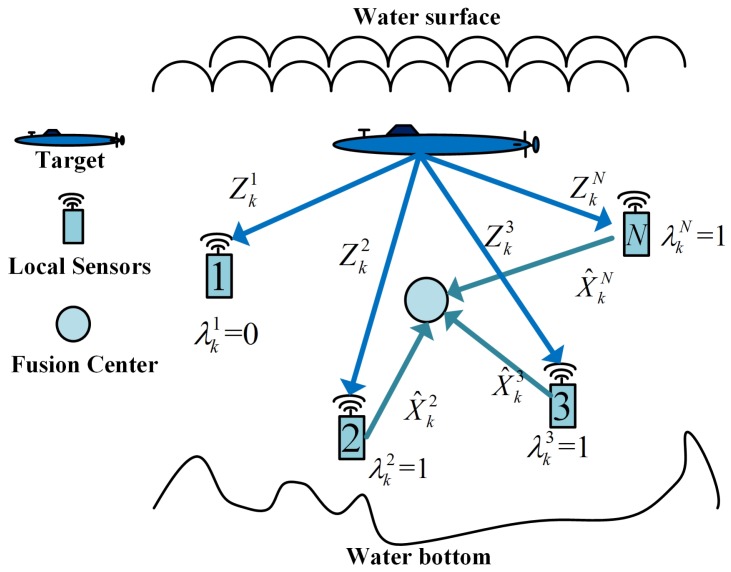
Measurement residual indicator based distributed fusion architecture.

The fusion center should formulate criteria to tell local sensors whether their estimates are needed or not. We adopt the standard proposed in [11] and make some changes in formulations for further convenience. We define an indicator function as

(7) $$\lambda_{k}^{i}=\left\{\begin{array}{c} 0,\;|{\tilde{Z}}_{k}^{i}|\leq \delta {E_{k}^{i}}^{T}\\ 1,\;\mathrm{otherwise} \end{array}\right.$$

where δ is the normalized threshold and the weight $E_{k}^{i}$ is determined by:

(8) $${E_{k}^{i}}^{T}E_{k}^{i}=H_{k}^{i}P_{k|k-1}{H_{k}^{i}}^{T}+R_{k}^{i},$$

where $H_{k}^{i}$ is the Jacobian matrix of the measurement function with the predicted estimate ${\hat{X}}_{k|k-1}$ and $P_{k|k-1}$ is the error covariance of ${\hat{X}}_{k|k-1}$. Once a local sensor *i* obtains fresh measurements at time *k*, it should calculate the corresponding indicator value $\lambda_{k}^{i}$ as Equation (7). If $\lambda_{k}^{i}=0$, sensor *i* needs to do nothing but keep silent to save energy. If $\lambda_{k}^{i}=1$, sensor *i* will calculate the local estimate and send it to the fusion center. Figure 2 illustrates how these indicator values work. It should be noticed that the measurement residual based fusion framework includes a feedback path from fusion center to local sensors. However, this feedback path adds to the negligible energy consumption of local sensors because the cost of receiving energy is much smaller than the cost of transmissitting energy according to [9].

## 4. Artificial Measurement Based Adaptive Filter

### 4.1. Artificial Measurement Model

Even if the fusion center did not receive local estimates from some local sensors, it obtained useful information that their measurement residuals are smaller than the threshold. For instance, if a fusion center did not get a packet from local sensor *i* at time *k*, then $\lambda_{k}^{i}=0$ and

(9) $${\hat{Z}}_{k|k-1}-\delta {E_{k}^{i}}^{T}\leq Z_{k}^{i}\leq {\hat{Z}}_{k|k-1}+\delta {E_{k}^{i}}^{T}.$$

Based on the well-known Bayesian formula, $Z_{k}^{i}$ conditioned on $\lambda_{k}^{i}=0$ and ${\hat{X}}_{k|k-1}^{i}$ has the distribution

(10) $$f\left(Z_{k}^{i}|\lambda_{k}^{i}=0,{\hat{X}}_{k|k-1}^{i}\right)\propto f\left(\lambda_{k}^{i}=0|Z_{k}^{i},{\hat{X}}_{k|k-1}^{i}\right)f\left(Z_{k}^{i}|{\hat{X}}_{k|k-1}^{i}\right).$$

where $f(\lambda_{k}^{i}=0|Z_{k}^{i},{\hat{X}}_{k|k-1}^{i})$ has the uniform distribution

(11) $$f\left(\lambda_{k}^{i}=0|Z_{k}^{i},{\hat{X}}_{k|k-1}^{i}\right)∼U\left(\left[{\hat{Z}}_{k|k-1}^{i}-\delta {E_{k}^{i}}^{T},{\hat{Z}}_{k|k-1}^{i}+\delta {E_{k}^{i}}^{T}\right]\right),$$

and before we get $\lambda_{k}^{i}$,

(12) $$E(Z_{k}^{i}-{\hat{Z}}_{k|k-1}^{i})=0,$$

(13) $$\mathrm{Cov}\left(Z_{k}^{i}-{\hat{Z}}_{k|k-1}^{i}\right)=H_{k}^{i}P_{k|k-1}{H_{k}^{i}}^{T}+R_{k}^{i}.$$

$f(Z_{k}^{i}|{\hat{X}}_{k|k-1}^{i})$ has the normal distribution as

(14) $$f\left(Z_{k}^{i}|{\hat{X}}_{k|k-1}^{i}\right)∼N\left({\hat{Z}}_{k|k-1}^{i},H_{k}^{i}P_{k|k-1}{H_{k}^{i}}^{T}+R_{k}^{i}\right).$$

So $f(Z_{k}^{i}|\lambda_{k}^{i}=0,\;{\hat{X}}_{k|k-1}^{i})$ is simply a truncated normal distribution as

(15) $$f\left(Z_{k}^{i}|\lambda_{k}^{i}=0,{\hat{X}}_{k|k-1}^{i}\right)∼N_{\left[a,b\right]}\left({\hat{Z}}_{k|k-1}^{i},H_{k}^{i}P_{k|k-1}{H_{k}^{i}}^{T}+R_{k}^{i}\right).$$

where $a={\hat{Z}}_{k|k-1}^{i}-\delta {E_{k}^{i}}^{T}$ and $b={\hat{Z}}_{k|k-1}^{i}+\delta {E_{k}^{i}}^{T}$. We do not want to drop above information about $Z_{k}^{i}$. Thus, we define an artificial measurement model as

(16) $${\bar{Z}}_{k}^{i}=Z_{k}^{i}+u_{k}^{i}.$$

where ${\bar{Z}}_{k}^{i}$ is the artificial measurement and $u_{k}^{i}$ is the zero-mean measurement noise. This model can be regarded as a measurement model of the real measurement and we want to use the artificial measurement to approximate the real measurement. The Equation (15) can be rewritten as

(17) $${\hat{Z}}_{k|k-1}^{i}=Z_{k}^{i}+\xi_{k}^{i}.$$

and

(18) $$\xi_{k}^{i}∼N_{\left[a,b\right]}\left(0,H_{k}^{i}P_{k|k-1}{H_{k}^{i}}^{T}+R_{k}^{i}\right).$$

It is obvious that Equation (17) matches our artificial measurement model such as Equation (16) well. If we let ${\hat{Z}}_{k|k-1}^{i}$ stand as the realization of our artificial measurement, the measurement noise $u_{k}^{i}$ has the same distribution as $\xi_{k}^{i}$. According to the characteristics of the truncated normal distribution, the variance of $u_{k}^{i}$ can be calculated as

(19) $$\mathrm{Var}\left(u_{k}^{i}\right)=\left(H_{k}^{i}P_{k|k-1}{H_{k}^{i}}^{T}+R_{k}^{i}\right)\left(1-\Delta \left(\delta \right)\right).$$

(20) $$\Delta \left(\delta \right)=2/\sqrt{2}\delta e^{-\delta^{2}/2}{\left[2\Phi \left(\delta \right)-1\right]}^{-1}.$$

where Φ(·) is the standard Φ-function defined by

(21) $$\Phi \left(\delta \right)=\int_{-\infty }^{\delta }1/\sqrt{2\pi }e^{-x^{2}/2}dx.$$

So our artificial measurement model Equation (16) has a unique realization ${\hat{Z}}_{k|k-1}^{i}$ and a truncated normal noise $u_{k}^{i}$.

### 4.2. Artificial Measurement Based Filter

Based on previous discussion about our artificial measurement model, we can derive our artificial measurement-based filter as follows

(1) *Predict*:

Assume that we already obtain the fusion estimate ${\hat{X}}_{k-1}$ and the corresponding error covariance $P_{k-1}$ at time *k*. The predicted state estimate and the corresponding error covariance at time *k* can be calculated as

(22) $${\hat{X}}_{k|k-1}=F_{k}{\hat{X}}_{k-1},$$

(23) $$P_{k|k-1}=F_{k}P_{k-1}F_{k}^{T}+Q_{k}.$$

(2) *Update*:

If $\lambda_{k}^{i}=1$, local sensor *i* can update the estimate and corresponding error covariance as EKF as follows.

Measurement residual:

(24) $${\tilde{Z}}_{k}^{i}=Z_{k}^{i}-h_{k}^{i}\left({\hat{X}}_{k|k-1}\right),$$

covariance of Measurement residual:

(25) $$S_{k}^{i}=H_{k}^{i}P_{k|k-1}{H_{k}^{i}}^{T}+R_{k}^{i},$$

where $H_{k}^{i}=H_{k}^{i}\left({\hat{X}}_{k|k-1}^{i}\right)$ refer to Equation (4). Optimal Kalman gain:

(26) $$K_{k}^{i}=P_{k|k-1}{H_{k}^{i}}^{T}{S_{k}^{i}}^{-1},$$

Updated state estimate and updated estimate covariance:

(27) $${\hat{X}}_{k}^{i}={\hat{X}}_{k|k-1}^{i}+K_{k}^{i}{\tilde{Z}}_{k}^{i},$$

(28) $$P_{k}^{i}=P_{k|k-1}^{i}-K_{k}^{i}H_{k}^{i}P_{k|k-1}^{i}.$$

If $\lambda_{k}^{j}=0$, the fusion center will not receive the local estimate of *j*, it can update the artificial local estimate and corresponding covariance itself with the help of the artificial measurement model as follows. Measurement residual:

(29) $${\tilde{\bar{Z}}}_{k}^{j}=h_{k}^{j}\left({\hat{X}}_{k|k-1}\right)-h_{k}^{j}\left({\hat{X}}_{k|k-1}\right)=0,$$

covariance of Measurement residual:

(30) $${\bar{S}}_{k}^{j}=\left(H_{k}^{j}P_{k|k-1}{H_{k}^{j}}^{T}+R_{k}^{j}\right)\left(2-\Delta \left(\delta \right)\right),$$

The above equation can be obtained by

(31) $$\begin{array}{cc} {\bar{S}}_{k}^{j} & =\mathrm{Cov}({\tilde{\bar{Z}}}_{k}^{j})\\ & =\mathrm{Cov}({\bar{Z}}_{k}^{j}-{\hat{Z}}_{k|k-1})\\ & =\mathrm{Cov}(Z_{k}^{j}-{\hat{Z}}_{k|k-1}+u_{k}^{j})\\ & =\mathrm{Cov}\left(Z_{k}^{j}-{\hat{Z}}_{k|k-1}\right)+\mathrm{Cov}\left(u_{k}^{j}\right)\\ & =H_{k}^{j}P_{k|k-1}{H_{k}^{j}}^{T}+R_{k}^{j}\\ & \;+\left(H_{k}^{j}P_{k|k-1}{H_{k}^{j}}^{T}+R_{k}^{j}\right)\left(1-\Delta \left(\delta \right)\right)\\ & =\left(H_{k}^{j}P_{k|k-1}{H_{k}^{j}}^{T}+R_{k}^{j}\right)\left(2-\Delta \left(\delta \right)\right), \end{array}$$

Optimal Kalman gain:

(32) $${\bar{K}}_{k}^{j}=P_{k|k-1}{H_{k}^{j}}^{T}{\bar{S}}_{k}^{j\;-1},$$

Updated state estimate and updated estimate covariance:

(33) $${\hat{X}}_{k}^{j}={\hat{X}}_{k|k-1}^{j}+{\bar{K}}_{k}^{j}{\tilde{\bar{Z}}}_{k}^{j}={\hat{X}}_{k|k-1}^{j},$$

(34) $$P_{k}^{j}=P_{k|k-1}^{j}-{\bar{K}}_{k}^{j}H_{k}^{j}P_{k|k-1}^{j}.$$

Finally, the fusion center will fuse these local estimates and artificial local estimates together to get the fusion estimate using the fusion algorithm proposed in [20].

(35) $$P_{k}={\left[\sum_{i=1}^{N}{P_{k}^{i}}^{-1}-\left(N-1\right)P_{k|k-1}^{-1}\right]}^{-1}.$$

(36) $${\hat{X}}_{k}=P_{k}\left[\sum_{i=1}^{N}{P_{k}^{i}}^{-1}{\hat{X}}_{k}^{i}-\left(N-1\right)P_{k|k-1}^{-1}{\hat{X}}_{k|k-1}\right].$$

**Figure 3:**
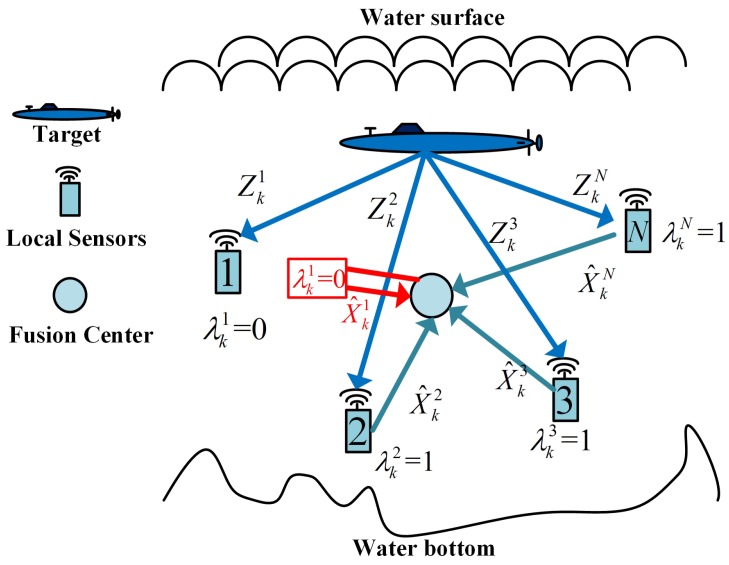
Artificial measurement-based distributed fusion architecture.

Figure 3 shows the structure of our artificial measurements-based distributed fusion filter. The red part tells us that the fusion center makes full use of the information of $\lambda_{k}^{1}=0$ and generates the artificial local estimate to compensate for the unsent real local estimate.

### 4.3. Adaptive δ Determination

The normalized threshold δ plays a key role in our artificial measurements based filter. Indicate function refereing to Equation (7), which is a function of δ, gives local sensors a criterion to decide whether to send local estimates to the fusion center or not. The larger the δ is, the lower probability local sensors have to send local estimates. That means δ affects the communication frequency of local sensors. In other words, δ can determine the energy consumption of local sensors. In addition, the estimate covariance in Equation (34) is a function of δ, which means δ affects not only the energy consumption but also the estimate accuracy of the target state. Both energy cost and target tracking accuracy are important in underwater target tracking issues. In this section, we proposes an adaptive δ determination method to make a better tradeoff between the communication cost and target tracking accuracy.

From Equations (7) and (8), we can obtain the probability distribution function of the indicator $\lambda_{k}^{i}$ as

(37) $$p\left(\lambda_{k}^{i}=0\right)=2\Phi \left(\delta \right)-1.$$

(38) $$p\left(\lambda_{k}^{i}=1\right)=2-2\Phi \left(\delta \right).$$

Consequently, the expectation of communication rate of local sensor *i* at time *k* is

(39) $$\begin{array}{cc} E(\lambda_{k}^{i}) & =2-2\Phi (\delta )\\ & =\mathrm{Energy}(\delta ). \end{array}$$

In this paper, we use the number of packets sent to the fusion center to measure energy cost at local sensors, which is defined as Energy(δ).

From Equations (28) and (34), we can calculate the expectation of estimate covariance of local sensor *i* at time *k* as

(40) $$\begin{array}{cc} E(P_{k}^{i})= & p\left(\lambda_{k}^{i}=0\right)\left(P_{k|k-1}^{i}-{\bar{K}}_{k}^{i}H_{k}^{i}P_{k|k-1}^{i}\right)\\ & +p\left(\lambda_{k}^{i}=1\right)\left(P_{k|k-1}^{i}-K_{k}^{i}H_{k}^{i}P_{k|k-1}^{i}\right). \end{array}$$

we rewrite above equation as

(41) $$E\left(P_{k}^{i}\right)=P_{k|k-1}^{i}+\mathrm{Error}\left(\delta \right)K_{k}^{i}H_{k}^{i}P_{k|k-1}^{i},$$

and

(42) $$\mathrm{Error}\left(\delta \right)=-[p\left(\lambda_{k}^{i}=0\right)/\left(2-\Delta \left(\delta \right)\right)+p\left(\lambda_{k}^{i}=1\right)].$$

It is clear that the estimate covariance increases with the increase of Error (δ). Equations (39) and (41) formulate how δ affects the energy costs of local sensors and target tracking accuracy. For the purpose of selecting proper δ during target tracking missions, we define an objective function as

(43) $$O\left(\delta \right)=\mathrm{Energy}\left(\delta \right)+\alpha_{k}\mathrm{Error}\left(\delta \right),$$

where $\alpha_{k}$ is a coefficient to adjust the weight of tracking error. The optimal δ can be determined by

(44) $$\delta^{opt}=\arg\;\min\;O\left(\delta \right).$$

Since we should guarantee target tracking performance first, the coefficient $\alpha_{k}$ should be large when tracking performance is bad. Conversely, $\alpha_{k}$ should be small if tracking accuracy meets our demand. In this paper, we use the trace of estimate covariance to measure tracking performance and $\alpha_{k}$ is determined as

(45) $$\alpha_{k}=\Theta_{k}/\Theta_{r},$$

where $\Theta_{k}$ is the trace of $P_{k}$ in Equation (35) and $\Theta_{r}$ is a pre-given reference value. It is not easy to get an analytical solution of Equation (44). Therefore, we provide an efficient numerical way to get the proper δ. Since δ is equivalent to the standard deviation of standard normal distribution, we select δ from 0 to 3 according to the well-known Pauta criterion. Then we take *n* uniformly-spaced samples from [0, 3] and form a set as

(46) $$\delta \in \{L_{1},\cdots ,L_{n-1},L_{n}\}.$$

Thus, Equation (44) can be rewritten as

(47) $$\delta^{opt}=\underset{\delta \in \{L_{1},\cdots ,L_{n-1},L_{n}\}}{\arg\;\min}O\left(\delta \right).$$

It should be noticed that $\mathrm{Energy}(L_{i})$ and $\mathrm{Error}(L_{i})$ can be calculated off-line to improve the online computational efficiency.

### 4.4. Optimal Sensor Group Selection

Assume that filtering results ${\hat{X}}_{k}$ and $P_{k}$ are given at time *k*. Then, target position at time k+1 can be predicted referring to Equation (1) and the distance $d_{k+1}^{i}$ from sensor *i* to the predicted target position can be calculated. Sensor *i* will have a chance to track the target and be a candidate sensor at time k+1 if $d_{k+1}^{i}$ is smaller than its sensing range. However, in 3D networks, we need four sensors to locate the position of a target [15], which means that adopting more than four sensors is not worthwhile if we consider their energy consumption. Thus, we select the best four sensors if there are more than four candidate sensors. Certainly, we employ all the candidate sensors when the number is less than or equal to four. We had proposed a posterior Cramer-Rao lower bound (PCRLB) based sensor selection scheme for particle filters in [21]. It calculated the PCRLBs of different sensor groups to evaluate how they contribute to tracking performance. However, in this paper, the fusion estimate covariance is given in Equation (35). That means we can evaluate how sensor groups contribute to tracking performance by $P_{k+1}$. This is more convenient than PCRLBs.

Given that we have a set of $N_{c}$ candidate sensors as $C=\{c_{1},c_{2},\cdots ,c_{N_{c}}\}$ at time k+1, the sensor selection problem can be formulated by

(48) $$G^{opt}=\underset{G\subset C}{\arg\;\min}\Psi_{k+1},$$

(49) $$\begin{array}{cc} \Psi_{k+1} & =\mathrm{Trace}(P_{k+1})\\ & =\mathrm{Trace}\{{\left[\sum_{i\in G}{P_{k+1}^{i}}^{-1}-\left(N-1\right)P_{k+1|k}^{-1}\right]}^{-1}\}, \end{array}$$

where $G=\{c_{g_{1}},c_{g_{2}},c_{g_{3}},c_{g_{4}}\}$ stands for sensor groups selected from set $\left\{c_{1},c_{2},\cdots ,c_{N_{c}}\right\}$. Equation (48) tells us that the sensor group that minimizes the trace of the fusion estimate covariance will be the best one. Since the rank of the traces of sensor groups in Equation (48) will not be changed with δ, $P_{k+1}^{i}$ is given by Equation (28) for simplified calculation. The exhaustive search is the most direct way to find the optimal sensor group and there will be $\frac{N_{c}!}{4!(N_{c}-4)!}$ groups. However, when $N_{c}$ is large, the number of the groups increase rapidly and the exhaustive search has a heavy computational burden. Therefore, we use the generalized Breiman, Friedman, Olshen and Stone (GBFOS) proposed in [22] to find the optimal sensor group. Initially, $N=N_{c}$ and the GBFOS algorithm keeps finding the optimal N−1 elements subset from the last optimal *N* elements set until N=4. Thus, GBFOS needs to try $\frac{\left(N_{c}-4\right)\left(N_{c}+5\right)}{2}$ sensor groups to find the optimal one. Table 2 lists some numerical examples to compare exhaustive search with GBFOS. It is obvious that GBFOS can reduce much more computational burden when $N_{c}$ becomes larger and larger.

We should mention the other search algorithm, called the greedy search, which is the reverse of GBFOS. The greedy algorithm keeps taking one optimal sensor out from candidate sensors until four sensors are taken out. Thus, the greedy search needs to try $\left(4N_{c}-6\right)$ sensor groups to find the optimal one. It seems better than GBFOS when $N_{c}$ is larger than 8. However, it is infeasible for our sensor selection scheme because there is no optimal sensor if we only choose one. That is according to

(50) $$\mathrm{Trace}\left(P_{k+1}^{i}\right)=\mathrm{Trace}\left(P_{k+1}^{j}\right),\;i,j\in C.$$

**Figure 4:**
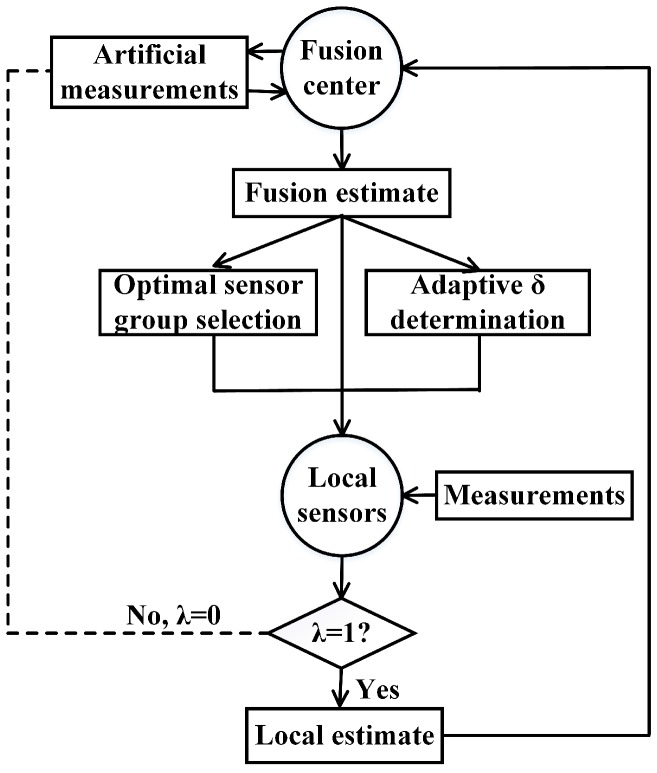
Flow chart of artificial measurements-based adaptive filter.

The flow chart of our artificial measurements-based adaptive filter is shown in Figure 4. It shows how information flows between the fusion center and local sensors. The dash line means the information flow from local sensors to the fusion center is non-existant when λ=0 and artificial measurements are introduced to compensate this missing information flow.

## 5. Simulation and Results

### 5.1. Simulation Scenario

We employ our artificial measurement-based filter to a target tracking mission for verification. In order to get more realistic performance measures, the target is assumed to move in a 3D underwater environment. The monitored field is 1000 m×1000 m×1000 m and sensors are deployed as a 5×5×5 uniform grid. All local sensors are identical. Their detection radius and measure covariance are 300 m and 10 m${}^{2}$, respectively. The initial state of the target is assumed to be ${\left[300,10,300,2,10,2\right]}^{T}$. From 1 s to 40 s, it moves at *constant velocity* (CV). From 41 s to 80 s, it makes a coordinate turn (CT) with turn rate 0.052 rad/s. From 81 s to 100 s, it moves at CV. CV and CT can be formulated as

(51) $$X_{k}=F^{\mathrm{CV}}\left(X_{k-1}\right)+w_{k},$$

(52) $$X_{k}=F^{\mathrm{CT}}\left(X_{k-1}\right)+w_{k},$$

where $F^{\mathrm{CV}}$ and $F^{\mathrm{CT}}$ are state transition matrixes. $w_{k}$ is the process noise with zero-mean white Gaussian distributions $N(0,Q_{k})$. $F^{\mathrm{CV}}$, $F^{\mathrm{CT}}$ and $Q_{k}$ are given by

(53) $$\begin{array}{c} \begin{array}{c} F^{\mathrm{CV}}=\left[\begin{array}{cccccc} 1 & T & 0 & 0 & 0 & 0\\ 0 & 1 & 0 & 0 & 0 & 0\\ 0 & 0 & 1 & T & 0 & 0\\ 0 & 0 & 0 & 1 & 0 & 0\\ 0 & 0 & 0 & 0 & 1 & T\\ 0 & 0 & 0 & 0 & 0 & 1 \end{array}\right], \end{array} \end{array}$$

(54) $$\begin{array}{c} \begin{array}{c} F^{\mathrm{CT}}=\left[\begin{array}{cccccc} 1 & \frac{\sin(wT)}{w} & 0 & \frac{\cos(wT)-1}{w} & 0 & 0\\ 0 & \cos(wT) & 0 & -\sin(wT) & 0 & 0\\ 0 & \frac{1-\cos(wT)}{w} & 1 & \frac{\sin(wT)}{w} & 0 & 0\\ 0 & \sin(wT) & 0 & \cos(wT) & 0 & 0\\ 0 & 0 & 0 & 0 & 1 & T\\ 0 & 0 & 0 & 0 & 0 & 1 \end{array}\right], \end{array} \end{array}$$

(55) $$Q_{k}=q^{2}\left[\begin{array}{cccccc} \frac{T^{3}}{3} & \frac{T^{2}}{2} & 0 & 0 & 0 & 0\\ \frac{T^{2}}{2} & T & 0 & 0 & 0 & 0\\ 0 & 0 & \frac{T^{3}}{3} & \frac{T^{2}}{2} & 0 & 0\\ 0 & 0 & \frac{T^{2}}{2} & T & 0 & 0\\ 0 & 0 & 0 & 0 & \frac{T^{3}}{3} & \frac{T^{2}}{2}\\ 0 & 0 & 0 & 0 & \frac{T^{2}}{2} & T \end{array}\right].$$

where *q* is the intensity of the process noise. For an underwater target, we consider that only on the *xoy* plane does it move as a CT model and it moves as a CV model in *Z*-axis direction.

### 5.2. Performance Verification

Simulation results are averaged over 100 Monte-Carlo runs. We adopt root mean square error (RMSE) to assess the accuracy of target tracking and the number of packets sent from local sensors to indicate the energy consumption.

#### 5.2.1. Performance Comparison

**Figure 5:**
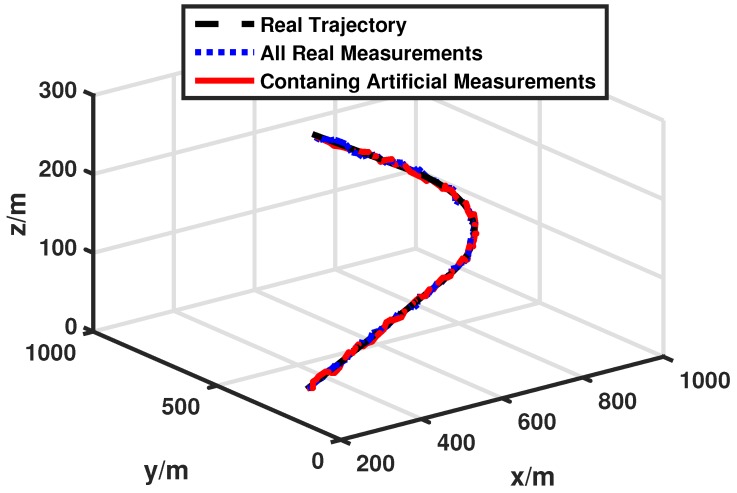
Target tracking performance: δ=0 versus δ=1.

**Figure 6:**
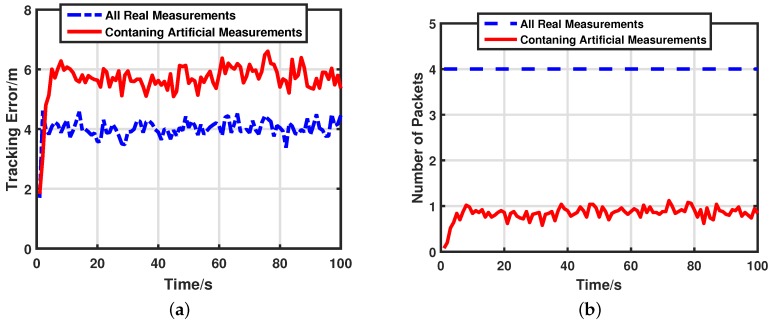
Performance comparison: δ=0 versus δ=1. (**a**) Target tracking error: δ=0 versus δ=1. (**b**) Energy consumptions: δ=0 versus δ=1.

In our simulation, we compare performances between the conventional target tracking scheme referring to δ=0 and our artificial measurements-based energy-efficient target tracking scheme with δ=1 to see how our algorithm achieves the goal of energy-efficiency. Figure 5 shows the real trajectory of the target and performances of different tracking schemes. All real measurements mean that the normalized threshold is equal to 0 and the fusion center has all local estimates. Containing artificial measurements means that the normalized threshold is not equal to 0 (in this case δ=1) and the fusion center updates the estimate with the help of artificial measurements. Both target tracking schemes can successfully track the target with high accuracy. The detailed tracking error and communication costs of the two schemes are displayed in Figure 6a,b respectively. They tell us that our target tracking scheme can save much energy (about 80%) while only loosing a small amount of tracking accuracy (about 40%). This is a worthwhile trade in the pursuit of saving energy.

#### 5.2.2. Impacts of δ

**Figure 7:**
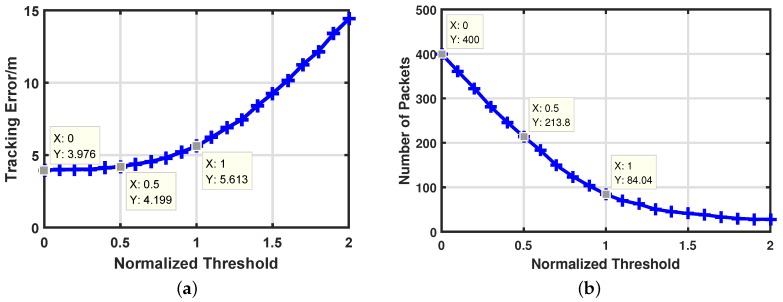
Impacts of normalized threshold δ. (**a**) Target tracking error with different δ. (**b**) Energy consumptions with different δ.

We also want to know how the normalized threshold δ affects the target tracking performance and communication costs of our algorithm. So we change the δ from 0 to 2 with the increment a of 0.1 at each step. Figure 7a illustrates the target tracking performance with different δ and we mark some points for further discussion. It is clear that target tracking error increases very slowly with a small δ. However, it increases faster and faster with the increase of δ. Compared with δ=0, it increases only 5.6% when δ=0.5 and 41.2% when δ=1. The error turns out to be extremely high (more than 300%) when δ=2. Correspondingly, the impact of different δ on communication costs is displayed in Figure 7b. Inversely, the communication rate falls rapidly when δ is small and it changes slowly when δ is lager than 1.5. The communication cost decreases by 46.5% when δ=0.5 and 79% when δ=1. Through Figure 7, we can find that our algorithm has a nice property with δ, which shows the potential that we can save much energy while only loosing a little tracking accuracy. Take δ=0.5 as a powerful example. We save 46.5% energy while loosing only 5.6% tracking accuracy. Therefore, the artificial measurements-based adaptive filter is proposed to find the proper δ.

#### 5.2.3. Performance of Adaptive Filter

**Figure 8:**
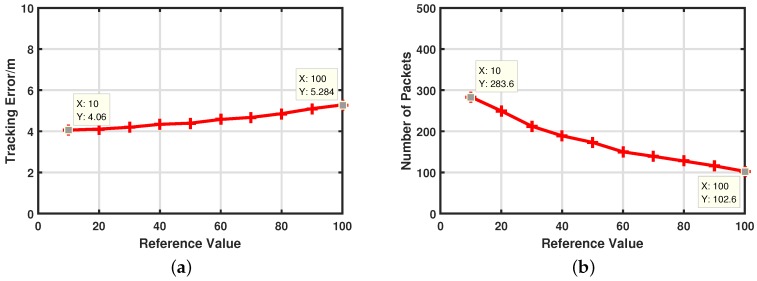
Impacts of pre-given reference value $\Theta_{r}$. (**a**) Target tracking error with different $\Theta_{r}$. (**b**) Energy consumptions with different $\Theta_{r}$.

Since the normalized threshold δ has opposite effects on target tracking accuracy and energy consumptions, it is important to find the proper δ to exploit the advantages of our artificial measurements-based filter. The core idea is to reduce energy consumption as much as possible under the premise of sufficient target tracking performance. In this paper, tracking performance is represented by $\alpha_{k}$ in Equation (45). A large $\alpha_{k}$ means tracking error is too large and we should put more effort into decreasing tracking error. In contrast, a small $\alpha_{k}$ means tracking performance meets our demand and we should pay attention to reducing energy cost. $\Theta_{r}$ in Equation (45) is a pre-given reference value to represent our demand for target tracking performance. In order to show the superiority of our artificial measurements-based adaptive filter, we change $\Theta_{r}$ from 10 to 100 with the increment of 10 at each step and set *n* in Equation (46) to 30. Figure 8a displays the target tracking performance with different $\Theta_{r}$. It is clear that target tracking error increases slowly with $\Theta_{r}$, which means our adaptive filter can guarantee target tracking performance even with a large $\Theta_{r}$. in contrast, the target tracking error becomes extremely high with a large δ in Figure 7a. Correspondingly, the impact of different $\Theta_{r}$ on communication costs is shown in Figure 8b. The communication cost decreases observably as $\Theta_{r}$ increases even with a small $\Theta_{r}$, which means our adaptive filter can effectively reduce energy consumption. Marked points in Figure 8 illustrate that our adaptive filter can exploit its advantages in energy saving and guarantee good target tracking performance. For example, compared with δ=0, we save 29.1% energy while lost only 2.11% tracking accuracy when $\Theta_{r}=10$ and we save 74.35% energy while loosing 32.9% tracking accuracy when $\Theta_{r}=100$. By setting different $\Theta_{r}$, the artificial measurements-based adaptive filter can achieve varying degrees of pursuits of energy saving.

#### 5.2.4. Performance of Sensor Group Selection

**Figure 9:**
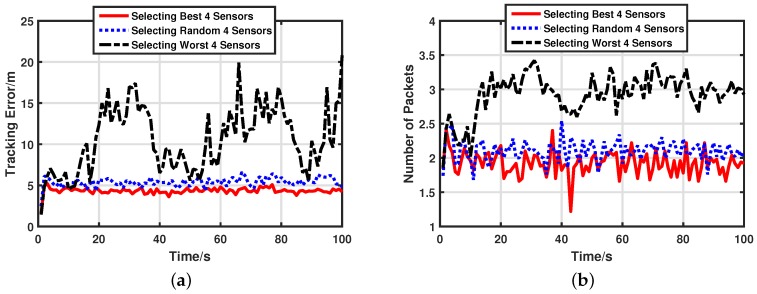
Performances of different sensor groups. (**a**) Target tracking error with different sensor groups. (**b**) Energy consumptions with different sensor groups.

Sensor group selection is a feasible method to improve the energy-efficiency of target tracking in UWSNs. We compare performances of three sensor group selection schemes to support this opinion. Here, we set $\Theta_{r}=40$. Target tracking error and energy consumptions of three schemes are displayed in Figure 9a,b, respectively. Selecting the best four sensors means that the sensor group is optimized using Equation (48). In contrast, selecting the worst four sensors means the sensor group is generated by

(56) $$G^{worst}=\underset{G\subset C}{\arg\;\max}\Psi_{k+1}.$$

Selecting a random four sensors means the sensor group is generated at random. Obviously, the performance of selecting the worst four sensors is much worse than other schemes. This must be avoided in terms of energy-efficiency and target tracking accuracy. The sensor group optimized by our sensor selection scheme has the best performance in both target tracking and energy saving. Average tracking accuracy and the total number of packets are listed in Table 3. Compared with the random sensor group, selecting the best four sensors can improve tracking accuracy by 19.61% and save 8.65% of energy.

**Figure 10:**
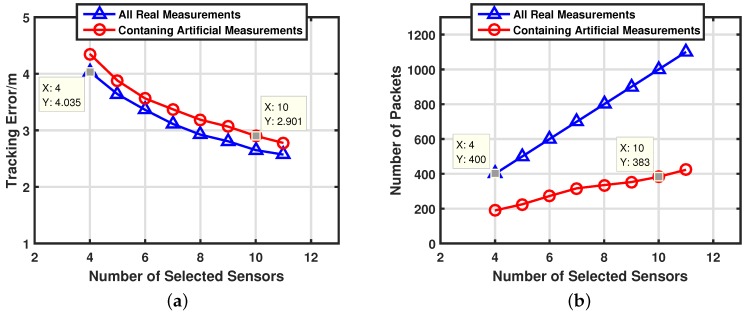
Performances of different number of selected sensors. (**a**) Target tracking error with different number of selected sensors. (**b**) Energy consumptions with different number of selected sensors.

Moreover, in some cases, we want to improve tracking accuracy with less additional energy consumption. This goal can be realized by selecting more sensors and using our artificial measurements-based adaptive filter. In order to have more candidate sensors, the sensors are deployed as a 7×7×7 uniform grid. The number of selected sensors $N_{s}$ at each step is changed from 4 to 11. We compare performances between artificial measurements-based adaptive filter with $\Theta_{r}=40$ and conventional all real measurements-based filter. The averaged target tracking error and communication costs of the two schemes are shown in Figure 10a,b respectively. It is clear that selecting more sensors can improve target tracking accuracy effectively with regard to both schemes. However, the energy consumption of an artificial measurements-based adaptive filter increases much more slowly than a conventional all real measurements-based filter. That means our target tracking scheme can improve target tracking performance with less additional energy consumption. Through marked points in Figure 10, the energy cost of our scheme with $N_{s}=10$ is 4.25% less than conventional scheme with $N_{s}=4$ and the target tracking error of our scheme with $N_{s}=10$ is 28.1% lower than conventional scheme with $N_{s}=4$. That means our scheme can have similar energy to a conventional scheme but get much better tracking performance than a conventional scheme.

**Figure 11:**
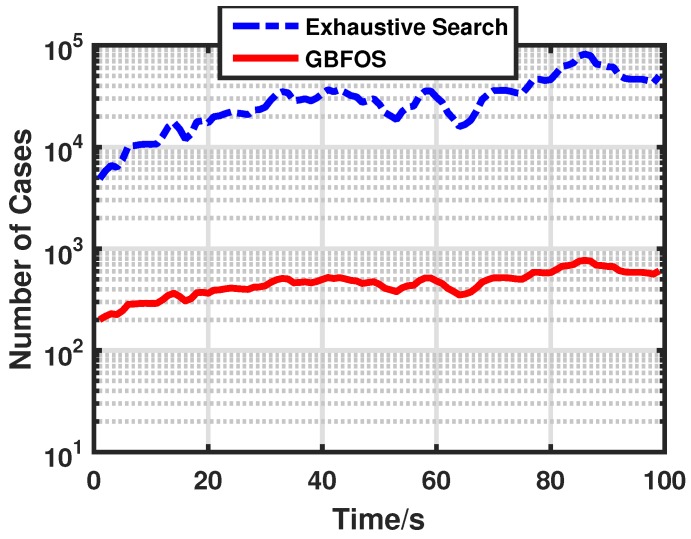
Number of cases needed to try of different search algorithms.

In a dense sensor network, the exhaustive search needs to try too many cases to find the best sensor group, which deteriorates the real-time performance of our optimal sensor group selection scheme. Hence, we use the GBFOS algorithm to reduce the number of cases and improve the computation efficiency of our optimal sensor group selection scheme. The number of cases needed to try to find the best sensor group of exhaustive search and GBFOS is plotted in Figure 11. Here we set $N_{s}=4$. Compared with the exhaustive search, the GBFOS algorithm reduces the number of cases by about two orders of magnitude. Hence, the GBFOS algorithm can remarkably improve the real-time performance of our optimal sensor group selection scheme.

Overall, this work focus on providing an energy-efficient target tracking algorithm for resource limited UWSNS. Our artificial measurements-based adaptive filter is easy to implement because it has widely applied the Kalman filter structure and low online computation demand.

## 6. Conclusions

This paper proposes an artificial measurements-based energy-efficient target tracking scheme in UWSNs. The basic idea of our approach is that, under the distributed fusion framework, we abandon low value local measurements to decrease the communication rate from local sensors to the fusion center to save energy. We guarantee the tracking accuracy by generating corresponding artificial measurements in the fusion center to compensate for unsent measurements. Then, we derive an adaptive filter based on these artificial measurements. In addition, we propose an optimal sensor selection scheme to further improve the energy-efficiency. Through simulation results, we can draw the following conclusions. Firstly, the artificial measurements based adaptive filter can save much energy while loosing less tracking accuracy. Secondly, by setting a different pre-given reference value $\Theta_{r}$, this adaptive filter can achieve varying degrees of pursuit of energy saving. Thirdly, our computationally efficient optimal sensor selection algorithm can efficiently improve target tracking performance under the premise of employing the same number of sensors. Finally, with the increase of the number of selected sensors, our artificial measurements-based adaptive filter better utilizes its advantages in energy-efficiency.

## Figures and Tables

**Table 1:** List of notations.

Notations	Explanations
$X_{k}$	Target state at time *k*
${\hat{X}}_{k}$	Estimate of target state at time *k*
${\hat{X}}_{k|k-1}$	Predicted estimate of target state at time *k*
$F_{k}$	State transition matrix at time *k*
$\omega_{k-1}$	Process noise at time k−1
$Q_{k}$	Covariance of process noise at time *k*
$Z_{k}^{i}$	Measurement of sensor *i* at time *k*
$\upsilon_{k}^{i}$, $u_{k}^{i}$, $\xi_{k}^{i}$	Measurement noise of sensor *i* at time *k*
$R_{k}^{i}$	Covariance of measurement noise of sensor *i* at time *k*
${\hat{Z}}_{k|k-1}^{i}$	Predicted measurement at time *k*
${\tilde{Z}}_{k}^{i}$	Measurement residual of sensor *i* at time *k*
${\bar{Z}}_{k}^{i}$	Artificial measurement of sensor *i* at time *k*
$h_{k}^{i}\left(\cdot \right)$	Measurement function of sensor *i* at time *k*
$H_{k}^{i}\left(\cdot \right)$	Jacobian matrix of sensor *i* at time *k*
$\left(x_{k},y_{k},z_{k}\right)$	Target location at time *k*
$\left(x_{i}^{s},y_{i}^{s},z_{i}^{s}\right)$	Location of Sensor *i*
δ	Normalized threshold
$\lambda_{k}^{i}$	Indicator value of sensor *i* at time *k*
${\hat{P}}_{k|k}$	Estimate error covariance at time *k*
${\hat{P}}_{k|k-1}$	Predicted estimate error covariance at time *k*
f(·)	Distribution of random variable
E(·)	Expectation of random variable
Cov(·)	Covariance of random variable
p(·)	Probability of random variable
$S_{k}^{i}$	Covariance of measurement residual of sensor *i* at time *k*
${\bar{S}}_{k}^{i}$	Covariance of measurement residual of sensor *i* at time *k* with artificial measurement
$K_{k}^{i}$	Kalman gain of sensor *i* at time *k*
${\bar{K}}_{k}^{i}$	Kalman gain of sensor *i* at time *k* with artificial measurement
$\Theta_{k}$	Trace of $P_{k}$
$\Theta_{r}$	Pre-given reference value

**Table 2:** The number of cases needed to try to find the best one.

	$N_{c}=6$	$N_{c}=8$	$N_{c}=10$	$N_{c}=15$	$N_{c}=20$
Exhaustive Search	15	70	210	1365	4845
GBFOS	11	26	45	110	200

**Table 3:** Performances of different sensor groups.

	Worst Sensor Group	Random Sensor Group	Best Sensor Group
Target tracking error	10.6308	5.3976	4.3389
Number of packets	292.65	210.34	192.16
